# A practical genome-enabled legitimacy assay for oil palm breeding and seed production

**DOI:** 10.1186/s12870-019-2062-x

**Published:** 2019-11-05

**Authors:** Chee-Keng Teh, Heng-Leng Lee, Hafiza Abidin, Ai-Ling Ong, Sean Mayes, Fook-Tim Chew, David Appleton

**Affiliations:** 1 Biotechnology & Breeding Department, Sime Darby Plantation R&D Centre, Banting, Selangor Malaysia; 2 School of Biosciences, University of Nottingham Malaysia, Semenyih, Selangor Malaysia; 3 School of Biosciences, University of Nottingham, Nottingham, UK; 4 Department of Biological Sciences, National University of Singapore, Lower Kent Ridge Rd, Singapore, Singapore

**Keywords:** Contamination, *SHELL*, DNA fingerprinting, Genetic purity, Seed quality control

## Abstract

**Background:**

Legitimacy in breeding and commercial crop production depends on optimised protocols to ensure purity of crosses and correct field planting of material. In oil palm, the presence of three fruit forms permits these assumptions to be tested, although only after field planting. The presence of incorrect fruit forms in a cross is a clear sign of illegitimacy. Given that *tenera* forms produce 30% more oil for the same weight of fruit as *dura*, the presence of low levels of *dura* contamination can have major effect during the economic lifespan of an oil palm, which is around 25 years. We evaluated two methods for legitimacy test 1) The use of SHELL markers to the gene that determines the shell-thickness trait 2) The use of SNP markers, to determine the legitimacy of the cross.

**Results:**

Our results indicate that the SHELL markers can theoretically reduce the major losses due to *dura* contamination of *tenera* planting material. However, these markers cannot distinguish illegitimate *tenera*, which reduces the value of having bred elite *tenera* for commercial planting and in the breeding programme, where fruit form is of limited utility, and incorrect identity could lead to significant problems. We propose an optimised approach using SNPs for routine quality control.

**Conclusions:**

Both *dura* and *tenera* contamination can be identified and removed at or before the nursery stage. An optimised legitimacy assay using SNP markers coupled with a suitable sampling scheme is now ready to be deployed as a standard control for seed production and breeding in oil palm. The same approach will also be an effective solution for other perennial crops, such as coconut and date palm.

## Background

Oil palm (*Elaeis guineensis* Jacq.) is an out-pollinating, but monoecious crop; however, maternal and paternal lineages for the purpose of seed production are developed independently based on presence/absence of kernel shell in the fruit. Thick-shelled *dura* palms produce lower oil yield than *tenera* heterozygotes, whereas shell-less *pisifera* palms are usually female sterile. The crosses between *dura* and *pisifera* result in thin-shelled *tenera* progenies that exhibit 30% higher oil yield than *dura* parents with full fertility restored. Hence, *tenera* palms are preferred for commercial planting.

Like other plant and animal breeding programmes, understanding the parentage of families and individuals is crucial. In oil palm, the goal is to measure combining ability between *dura* and *pisifera* based on performance of their *tenera* progenies, also known as progeny testing. Controlled pollination is required to ensure the test reflects the true families or backgrounds. However, pollination errors, such as a contaminated pollen source, inflorescence bag damage and late bagging are still evident in oil palm breeding and seed production, leading to illegitimacy [1]. Aside from instances of uncontrolled pollination, hermaphrodite flowers occasionally form whereby self-pollination can occur [2], although the bunches with high number of male inflorescences can usually be identified and removed. Hence, seed illegitimacy in oil palm can happen at any stage from pollen collection, pollination and seed processing through to field planting. Quality assurance and problem identification are usually difficult, especially when combinatorial errors occur.

To date, the oil palm industry still relies on the shell fruit form and the presence of a fiber ring around the kernel to determine genetic purity of seed lots in breeding and commercial seed production. Seeds derived from *dura* (*Sh + Sh+*) x *pisifera* (*sh- sh-*) are theoretically 100% *tenera* (*Sh + sh-*) due to co-dominant monogenic inheritance [3]. Unexpected *dura* and *pisifera* progeny palms are defined as non-*tenera* contamination. However, the fruit census can only be carried out 2–3 years after field planting. Census methods based on a cross-section of mature fruit is laborious and inefficient. Most importantly, replacement of contaminants at this mature stage is not economically viable, leading to long-term yield loss from these contaminant palms throughout the 20–25 years of production lifespan. To solve this issue, novel mutations in the *SHELL* gene that are responsible for the fruit form have been identified [4] and can be used for detecting non-*tenera* contamination as early as the seed stage. Early elimination of the contaminants before field planting would significantly improve cost effectiveness and reduce yield losses due to poor seed production quality, hence improving oil palm productivity per unit area of land [5, 6]. However, conventional fruit census and SHELL testing both share two key limitations, whereby the methods are unable to identify illegitimate *tenera* that are derived from the wrong crossing or parentage and do not provide informative data as to the production causes of the illegitimacy. The wrong parentage illegitimacy is believed to have further impact on oil yield due to the different genetic combining ability of every resulting progeny seed produced from a pollinated flower. This is especially impactful in breeding programmes where illegitimate parental stock can effect multiple future generations and confuse interpretations from breeding trials.

Established seed producers and plantation companies have dedicated breeding programmes to sustain their planting requirements. Accurate pedigree records enable these producers to utilize legitimacy tests on every cross or seed lot before releasing the seeds to stakeholders, which often include smallholder plantations and farmers. The objective of this study was to develop an improved practical assay for legitimacy test using an optimised number of genomic single nucleotide polymorphism (SNP) markers, followed by implementation as a commercial quality control tool. A commercial population of *dura*, half- and full-sib mother palms was blind tested to determine the accuracies of legitimacy test using 1000 SNPs and marker subsets optimised.

## Results

### Genetic clustering and fruit form validation

**Fig. 1:**
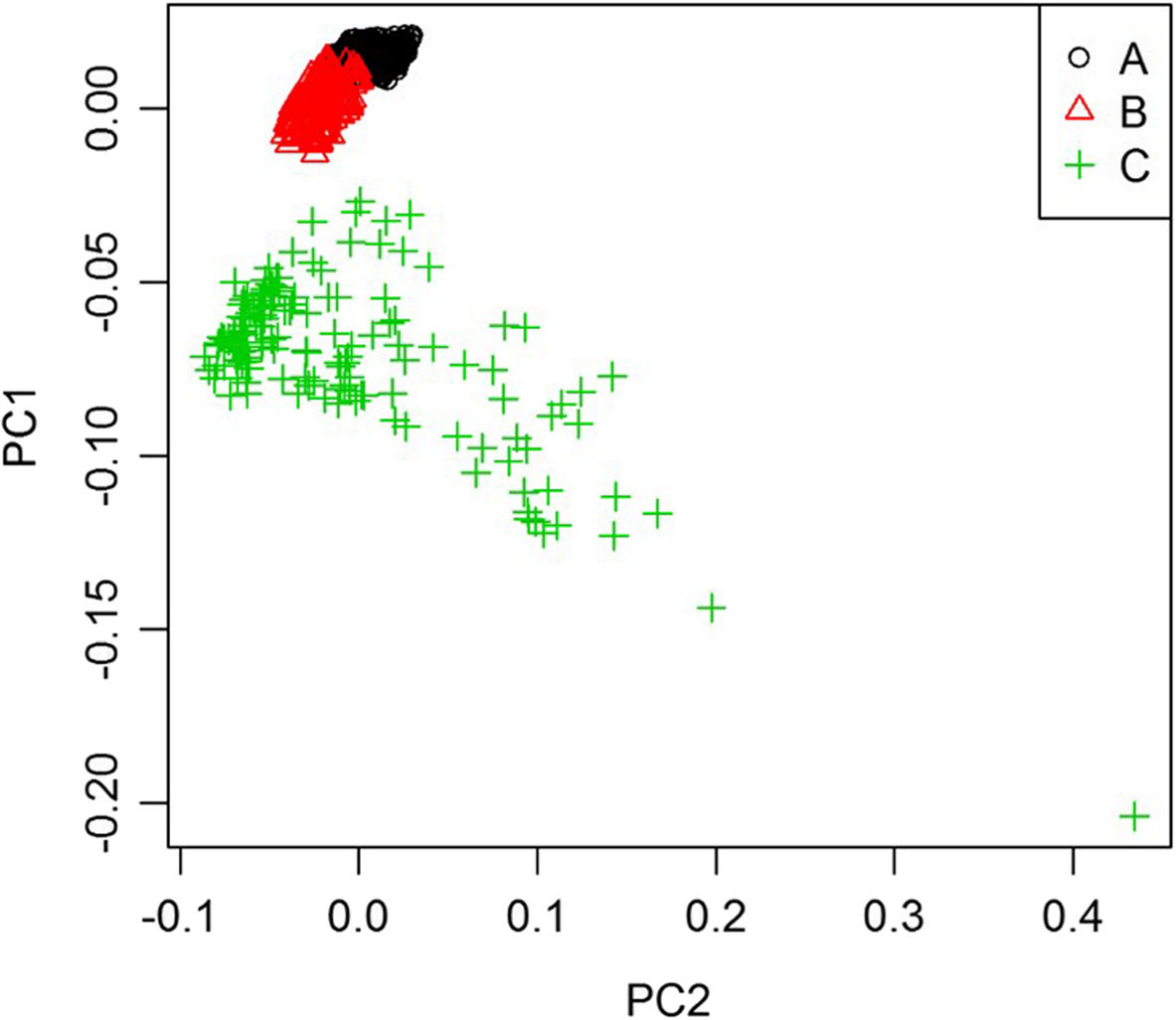
Genetic clustering of 1000 oil palm samples. Three clusters including A – Family A (Deli *Dura* 1 x AVROS *Pisifera* 1), B – Family B (Deli *Dura* 2 x AVROS *Pisifera* 1) and C – Family C (Deli *dura* palms). PC – principal component

The assayed SNPs had < 5% missing data in the GS1000™ panel for 988 markers. The markers successfully assigned the 1000 palms to three clusters i.e. Family A (700 palms; black), Family B (150 palms; red) and Family C (150 palms; green) in a principle component plot (Fig. 1). Family A and Family B were more genetically related due to their half-sib lineage and were therefore overlapping. A broader distribution of Family C was due to mother palms derived from different lineages. In addition, the fruit forms of all three families were also validated by the SHELL test. Family A and Family B with *sh*^*Deli*^*/sh*^*AVROS*^ genotype were *tenera*, whereas Family C with *sh*^*Deli*^*/sh*^*Deli*^ were *dura*. Overall, the result indicated a good agreement with known pedigree records and observed fruit form.

### Legitimacy reference of Family A

**Fig. 2:**
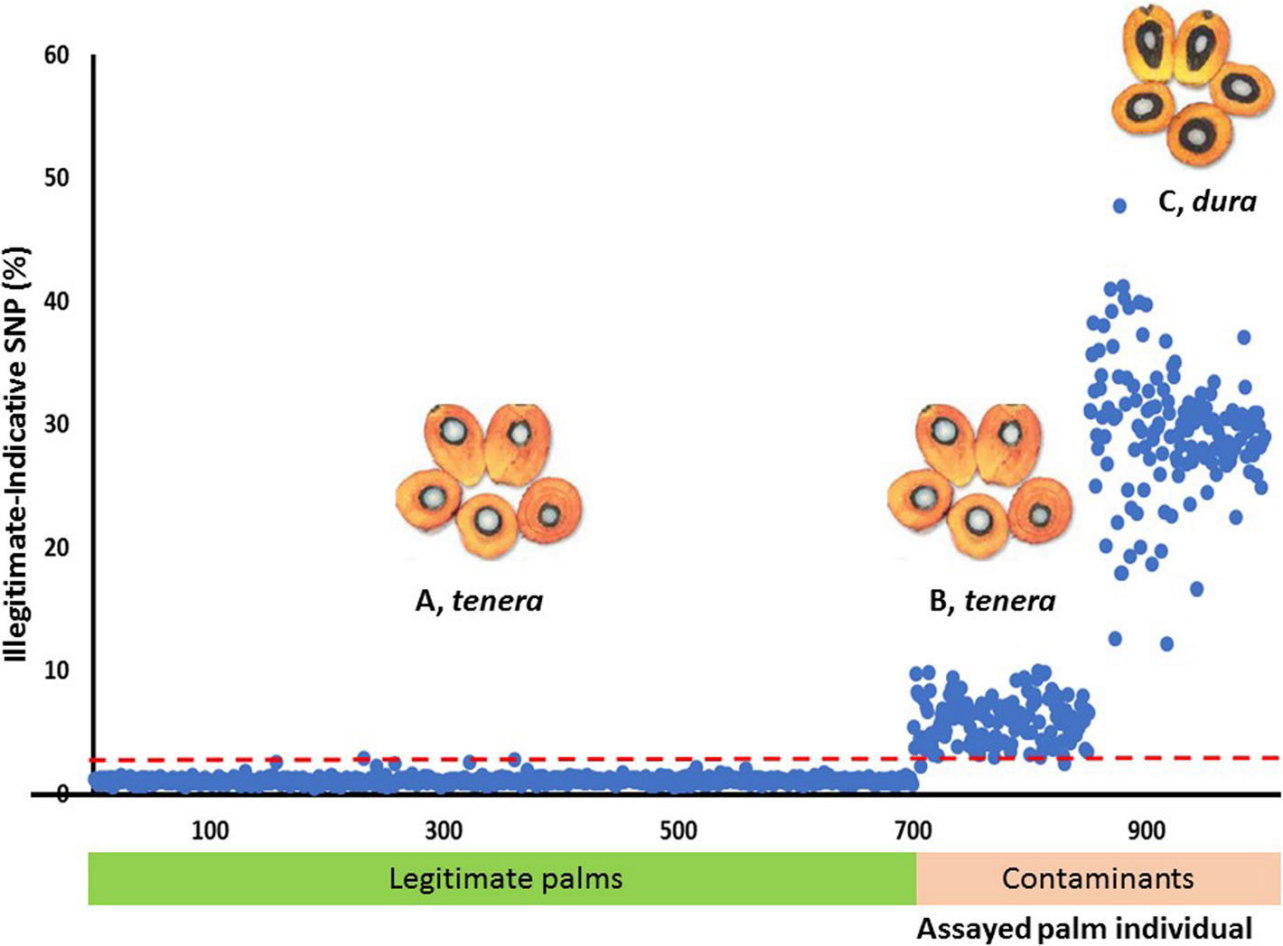
Legitimacy reference based on the parentage of Family A at illegitimacy-indicative SNP threshold = 3% (the dotted line in red). A = Family A (Deli *Dura* 1 x AVROS *Pisifera* 1), Family B (Deli *Dura* 2 x AVROS *Pisifera* 1) and Family C (Deli *dura* palms). Each blue dot signifies a tested seed

Family A exhibited 0.5–2.9% illegitimacy-indicative SNPs and therefore can be defined as a legitimate population. The two illegitimacy sources were successfully resolved using the GS1000™ panel (Fig. 2). All *dura* contaminants from Family C were clearly distinguished with 12.3–47.7% illegitimacy-indicative SNPs. The second contaminant source, Family B was 98.7% half-sib *tenera* with 3.0–10.0% illegitimacy-indicative SNPs. This information was used as a legitimacy reference standard for subsequent sensitivity analysis.

### Sensitivity of legitimacy test across marker densities

**Fig. 3:**
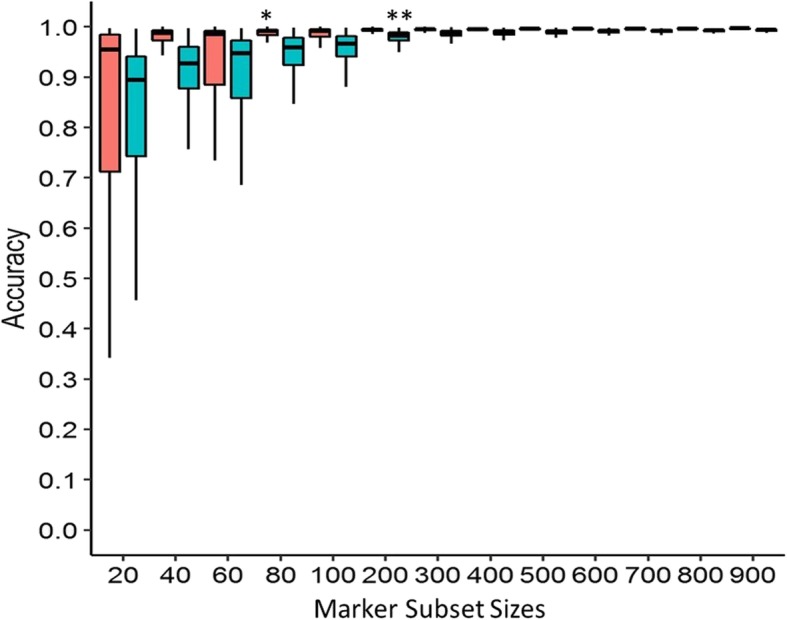
Sensitivity of legitimacy test across marker subset sizes. Red box = Family (A + C); Blue box = Family (A + B + C). * The plateau point is at 80 SNPs with 0.959 accuracy mean (± 0.096) for Family (A + C). ** The plateau point is at 200 SNPs with 0.977 accuracy mean (± 0.027) for Family (A + B + C). Family (A + C) indicates occurrence of *dura* contamination only and Family (A + B + C) indicates occurrence of both *dura* and *tenera* contamination

In all family combinations, the accuracy improved significantly when more markers were used. Family (A + C) and Family (A+ B + C) had the highest accuracy mean, recorded 0.997 (± 0.001) and 0.993 (± 0.002) at 900 SNPs, respectively (Fig. 3). However, the accuracy plateaued at different marker subset sizes depending on the family combinations. For Family (A + C), the plateau point was at 80 SNPs with 0.959 accuracy mean (± 0.096). The plateau point of Family (A + B + C) was at 200 SNPs with 0.977 accuracy mean (± 0.027).

## Discussion

For perennial species such as oil palm, selective breeding of elite *dura* and *pisifera* individuals is based on phenotypic performance of their progenies (reflected in combining ability). Legitimacy of a commercial progeny is declared if non-*tenera* contamination through fruit census is lower than 5%. Nevertheless, excessive *dura* and *tenera* can be commonly found in breeding crossing programmes used to expand and improve parental lines, such as *dura* x *tenera* and *tenera* x *tenera*, causing significant deviation from theoretical Mendelian segregation ratios (Chi-square test) [7]. This suggests that ‘hidden’ illegitimacy can exist at unknown levels, where shell-type is expected to segregate or even where shell-type is consistent with legitimacy, as in commercial *tenera* material.

**Fig. 4:**
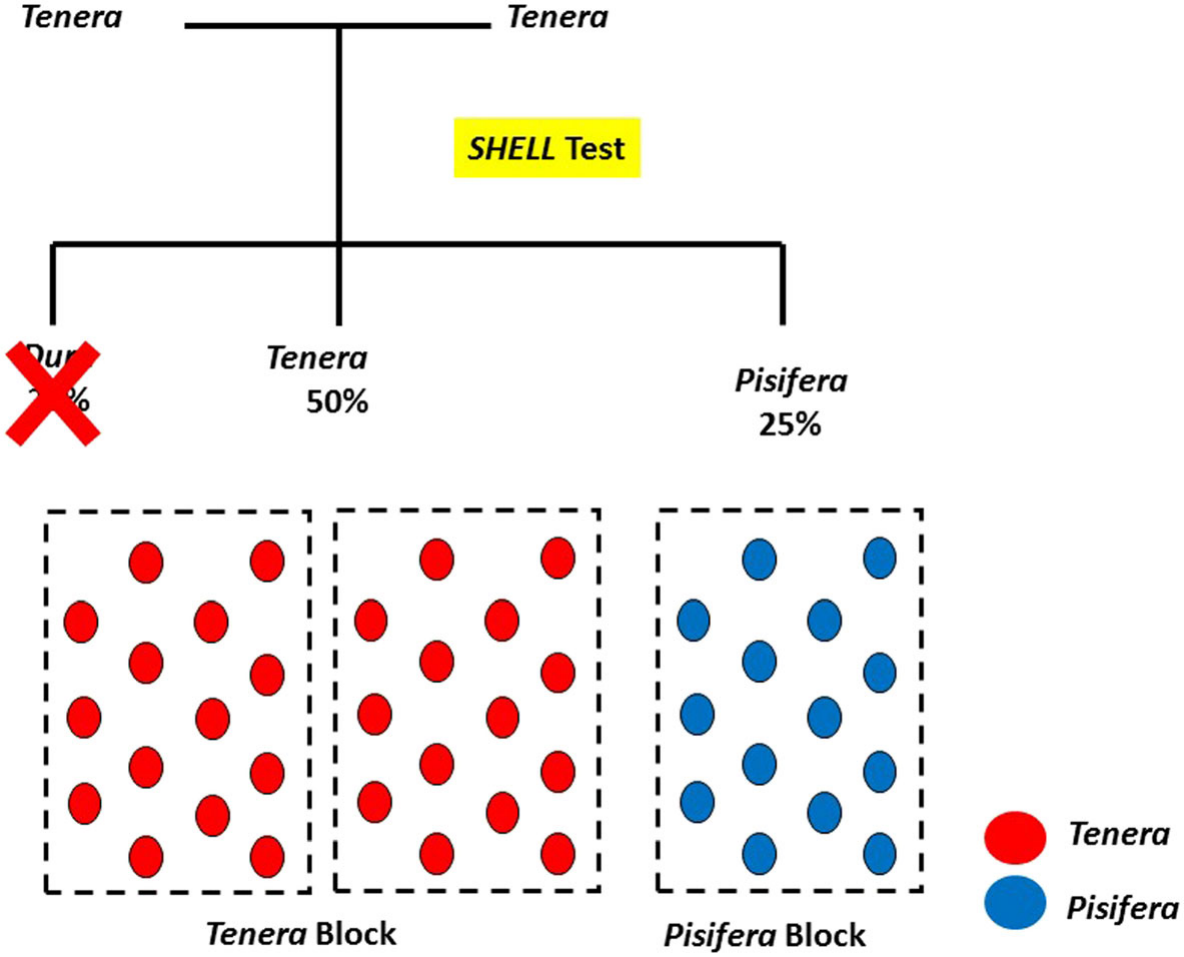
Application of SHELL markers in *a tenera* x *tenera* cross for *pisifera* selection programmes. The SHELL test enables differentiation of three fruit forms i.e. *dura*, *tenera* and *pisifera* at nursery stage. *Tenera* and *pisifera* with the desired number of palms can be planted in different blocks and 25% of trial land area can be reduced by removing the unwanted *dura*

Estimating 1% *dura* contamination may incur a cost of RM 178.8 million/year (USD 45.6 million/year) based on 28.6 million MT annual crude palm oil (CPO) production in Malaysia [8], given a 25% oil loss yield from *dura* fruits compared with *tenera* fruits and a CPO price of RM 2500/MT (USD 638/MT). Hence, the importance of detection of fruit form before planting using SHELL markers becomes more evident when considering the reported average of 10.7% non-*tenera* contamination in Malaysia’s smallholder plantings [6]. The SHELL test also immediately benefits the *pisifera* selection programme. Due to common female sterility, new *pisifera* parents are derived using sib-mating i.e. *tenera* x *tenera* or *tenera* x *pisifera*. In a *tenera* x *tenera* crosses, the fruit form segregation of the progeny usually follows a Mendelian ratio of 1 *dura*: 2 *tenera*: 1 *pisifera* (Fig. 4). The oil palm breeders can remove unwanted *dura* seedlings through the SHELL test to reduce 25% of total land used for a trial, leaving the desired number of *tenera* and *pisifera* to be planted in discrete blocks for easier trial management and data recording in the future. As *pisifera* are usually female sterile, they tend to allocate more resources to vegetative growth and often compete strongly with *dura* and *tenera* palms in the same trial, leading to a potential reduction in performance of the fruit forms with shells. The breeders can also conduct yield evaluation and pollen collection in the separated blocks, instead of spending time tracking the palms in the conventional random planting.

In this study, a total of 1000 palms were successfully assayed using the GS1000™ SNP panel and assigned to three clusters according to their pedigree i.e. Family A, Family B and Family C (Fig. 1). The results further validated the utility of the OP200K genotyping array used in previous studies [9, 10]. Family A and Family B which overlapped, were indeed half-sibs with a common paternal parent, AVROS *Pisifera* 1. This explains the higher genetic relatedness between these two families. Family C originated from multiple lineages of *dura* mother palms and could be clearly distinguished in the principle component plot, indicating the distinct genetic base. A legitimacy reference based on the parentage of Family A was then established (Fig. 2). At a 3% illegitimacy-indicative SNP threshold, for *dura* contamination (Family C) was distinguished through legitimacy test. Although the *sh*^*AVROS*^ mutation alone can explain 100% of the fruit form variation in the AVROS-based *tenera* palms in this study, this may not be the case in other origins due to additional reported novel mutations in the *SHELL* gene [4, 6]. Further characterisation on the haploinsufficiency of the *SHELL* gene also unveiled possible novel mutations and the potential for cis-compound mutations yet to be characterised. This may confound shell-type prediction, particularly in introgression hybrids and more exotic germplasm [11]. Legitimacy tests based on identity by descent can effectively address this issue. The legitimacy test developed in this study was also able to distinguish the *tenera* fruit of Family A and Family B, while SHELL markers did not distinguish them and identified them correctly as both 100% *tenera*. With 0% *dura* occurrence, the current Standards and Industrial Research Institute of Malaysia (SIRIM) certification for commercial seed production would declare the mix of Family A and B as fully legitimate, but part of the sample set was actually from incorrect parentage (Family B). The yield loss due to *tenera* contamination can now be prevented or at least accounted for with the help of this practical legitimacy assay protocol. The same scenario occurs in other crops whereby illegitimate individuals cannot always be distinguished by phenotype. For example, in coconut (*Cocos nucifera* L.) breeding, the petiole colour is often used as phenotypic marker to determine genetic purity of Yellow Dwarf x Tall hybrids, but no colour variation is observed within Tall cultivars [12].

The DNA fingerprinting technique was developed in the 1980’s after discovering restriction fragment length polymorphism (RFLP) markers in human DNA [13]. More marker systems were then discovered and applied in mammals [14], birds [15] and eventually plants. To increase genotyping throughput and polymorphism, oil palm researchers shifted their preference to microsatellite markers and the results were promising [16–18]. The technique has been strongly recommended as standard practice in all crosses and for tissue culture clones for the past decade. However, full adoption of legitimacy fingerprinting for commercial seed production has not been previously realized, mainly due to high assay cost and unclear sampling method. To address this, a practical method using SNP markers was developed for commercial-scale legitimacy test and the marker set size was optimised down from 1000 to 200 loci, sufficient to identify both *dura* and *tenera* contamination with consistent accuracy based on the test Family (A + B + C) (Fig. 3). Interestingly, only 80 loci were required to distinguish *dura* contaminants with consistent accuracy based on Family (A + C). An oil palm seed producer usually produces more than a million seeds annually. Testing every seed per cross is impractical and economically unjustifiable. However, many established sampling schemes for quality control, such as ISO2859-3 series are widely adopted in the manufacturing sector. These schemes provide a comprehensive reference on effective sample size to achieve the quality acceptance level based on the available seed lot and can also be adopted effectively to provide a quality underpinning to the legitimacy assay developed here for seed production quality control. Re-evaluation of the assay, however, is still necessary when dealing with alternative populations, or if the genetic base of existing parent populations becomes narrow due to selective breeding.

## Conclusion

Legitimacy tests using SNP markers can identify both *dura* and *tenera* contamination at or before the nursery stage. An optimised marker set of 200 SNPs coupled with a suitable sampling scheme will enable implementation of legitimacy test with accuracy more than 97% as a standard quality control procedure for seed purity in breeding and commercial production. Without access to pedigree records and parent palms, the SHELL test is still a powerful tool for the smallholders to discard *dura* contaminations before field planting. For perennial crops such as oil palm, illegitimacy can easily result in failure of a 12-year breeding selection cycle, and for commercial palms, a 25-year reduction in yield potential. Hence, genetic purity and good agricultural practices are equally essential to ensure the highest oil productivity of the oil palm industry into the future.

## Methods

### Plant materials and DNA preparation

A total of 1000 palms comprising of Family A (700 palms from Deli *Dura* 1 x AVROS *Pisifera* 1), Family B (150 palms from Deli *Dura* 2 x AVROS *Pisifera* 1) and Family C (150 Deli *dura* palms) was selected. Family A and Family B were produced as commercial *tenera* seeds and Family C was used as mother palms for breeding and commercial seed production. Also, the three parent palms, known as Deli *Dura* 1, Deli *Dura 2* and AVROS *Pisifera 1* were included. All palms were derived from in-house breeding materials and maintained at Sime Darby Plantation R&D Centre, Malaysia. Total genomic DNA was isolated from 0.1 g of young leaf tissue (at frond 0) using the DNAeasy Plant Mini Kit (Qiagen, Germany).

### SNP genotyping

The 1003 palms were genotyped for the legitimacy test and fruit form validation. A total of 1000 SNPs were selected from the OP200K Infinium array (Illumina) [9] to form a smaller genotyping panel, namely GS1000™. The probe design for the SNPs was done based on the requirement of the Kompetitive Allele Specific PCR™ (KASP™) genotyping platform. About 0.3 ng of the genomic DNA was used as template. Two fluorophores FAM and HEX were used to distinguish the KASP™ genotyping data. In the clustering plot, the samples marked in red and blue were homozygous for alleles with HEX and FAM, respectively. The heterozygous samples appeared in green. To confirm the fruit forms of assayed palms, the same genotyping method was done using KASP™ probes for *sh*^*mpob*^ and *sh*^*AVROS*^ mutations [11] at their reported genomic positions, i.e. 3,078,161 bp and 3,078,154 bp on Chromosome 2 [4]. Only the SNPs with lower than 5% missing data were selected for subsequent analysis.

### Data analysis

Genetic clustering among three families (A, B and C) was analysed in the R Package *SNPRelate* with default parameters [19]. The SHELL test and genetic relatedness among the assayed palms were used to validate their pedigree records. The legitimacy test was then performed using an in-house developed programme, namely *LegiPerl 1.0*. The programme classified samples as legitimate only if the progeny and parental genotypes followed Mendelian inheritance (Table 1). In this test, the ‘legitimate’ individuals accepted ≤3% of illegitimacy-indicative SNPs. The KASP™’s average genotyping error in positive DNA samples was reported to be 0.7–1.6% [20].

**Table 1:** Progeny and parental genotypes that follow Mendelian inheritance

*Dura* Parent		*Pisifera* Parent	*Tenera* Progeny
*A/A*	*X*	*a/a*	*A/a*
*A/A*	*X*	*A/a*	*A/A, A/a*
*A/a*	*X*	*A/A*	*A/A, A/a*
*A/a*	*X*	*A/a*	*A/A, A/a, a/a*

*A/A* and *a/a* = homozygous genotypes; *A/a* = heterozygous genotypes

For the blind test, Family B and Family C were assumed to be contaminants for Family A. The legitimacy tests were conducted in different combinations of families, reflecting possible contamination types in production/field: (i) Family (A + C) (850 palms) for *dura* contamination, (ii) Family (A + B) (850 palms) for half-sib contamination, and (iii) Family (A + B + C) (1000 palms) for multiple contamination sources. A legitimacy reference was constructed from Family (A + B + C) using the GS1000™ panel. This was followed by sensitivity analysis of the legitimacy test using different SNP subsets ranging from 20 to 900 markers with stepwise increments of 20 up to 100 SNPs and then 100 markers up to 900 for each combination of (i) and (iii). Each marker subset was analysed with 1000 iterations of random marker selection from the GS1000™ panel. The accuracy of each test was then determined based on the observed number of illegitimate palms that could not be distinguish from the reference. The descriptive statistics and graph plotting for accuracy were generated in R.

## Data Availability

The datasets used and/or analysed during the current study are available from the corresponding author on reasonable request.

## Abbreviations

CPO: Crude palm oil
KASP™: Kompetitive Allele Specific PCR™
RFLP: Restriction fragment length polymorphism
SIRIM: Standards and Industrial Research Institute of Malaysia
SNP: Single nucleotide polymorphism
